# Mathematical modeling of the synergistic interplay of radiotherapy and immunotherapy in anti-cancer treatments

**DOI:** 10.3389/fimmu.2024.1373738

**Published:** 2024-05-08

**Authors:** Paolo Castorina, Filippo Castiglione, Gianluca Ferini, Stefano Forte, Emanuele Martorana, Dario Giuffrida

**Affiliations:** ^1^ Genomics and molecular oncology unit, Istituto Oncologico del Mediterraneo, Viagrande, Italy; ^2^ Istituto Nazionale di Fisica Nucleare, Sezione di Catania, Catania, Italy; ^3^ Faculty of Mathematics and Physics, Charles University, Prague, Czechia; ^4^ Biotech Research Center, Technology Innovation Institute, Abu Dhabi, United Arab Emirates; ^5^ Institute for Applied Computing, National Research Council of Italy, Rome, Italy; ^6^ Radiotherapy Unit, REM Radioterapia, Viagrande, Italy; ^7^ School of Medicine, University Kore of Enna, Enna, Italy

**Keywords:** mathematical modeling, Gompertz law, radiotherapy, immune response, abscopal effect, immunotherapy

## Abstract

**Introduction:**

While radiotherapy has long been recognized for its ability to directly ablate cancer cells through necrosis or apoptosis, radiotherapy-induced abscopal effect suggests that its impact extends beyond local tumor destruction thanks to immune response. Cellular proliferation and necrosis have been extensively studied using mathematical models that simulate tumor growth, such as Gompertz law, and the radiation effects, such as the linear-quadratic model. However, the effectiveness of radiotherapy-induced immune responses may vary among patients due to individual differences in radiation sensitivity and other factors.

**Methods:**

We present a novel macroscopic approach designed to quantitatively analyze the intricate dynamics governing the interactions among the immune system, radiotherapy, and tumor progression. Building upon previous research demonstrating the synergistic effects of radiotherapy and immunotherapy in cancer treatment, we provide a comprehensive mathematical framework for understanding the underlying mechanisms driving these interactions.

**Results:**

Our method leverages macroscopic observations and mathematical modeling to capture the overarching dynamics of this interplay, offering valuable insights for optimizing cancer treatment strategies. One shows that Gompertz law can describe therapy effects with two effective parameters. This result permits quantitative data analyses, which give useful indications for the disease progression and clinical decisions.

**Discussion:**

Through validation against diverse data sets from the literature, we demonstrate the reliability and versatility of our approach in predicting the time evolution of the disease and assessing the potential efficacy of radiotherapy-immunotherapy combinations. This further supports the promising potential of the abscopal effect, suggesting that in select cases, depending on tumor size, it may confer full efficacy to radiotherapy.

## 1 Introduction

Immunological experiments during the last two decades have answered many important questions related to the causal relationship between chronic inflammation and carcinogenesis. The presence of inflammatory cells in the cancer milieu raises the question of the tumor progression despite a likely immune system reaction to tumor antigens. This aspect is particularly important since untreated tumors grow according to non-linear, macroscopic, laws as the Gompertz law (GL) Gompertz (1); Norton (2); Vaghi et al. (3) or the logistic one (LL) Verhulst (4); Vaghi et al. (3). Therefore those growth patterns emerge, at a larger level of magnification, from many microscopic biological factors, which turn out to be summarized by simple mathematical descriptions.

Since prolonged inflammation is a hallmark of cancer Hiam-Galvez et al. (5), initiating tumor genesis or supporting tumor growth, and the global immune response is significantly altered during tumor progression, immunotherapy is becoming a valid option in cancer treatment. However, the immune response can be detrimental rather than helpful [see, for example, Lin et al. (6)]: individual auto-antibodies play an antagonist role in cancer, but the agonist auto-antibody in some cancer patients turned out to be deleterious and harmful.

Some preclinical and clinical evidence confirm the synergistic action of radiotherapy (RT) and immunotherapy against the tumor cells Zhao and Shao (7). Although The intrinsic sensitivity to radiation is patient-specific Puglisi et al. (8); Puglisi et al. (9) and may depend on different factors, RT is able to ablate cancer cells not only by directly induced necrosis or apoptosis but also by triggering an immune response that actively recruits immune cells within the tumor microenvironment. For example, RT promotes the release of tumor-associated antigens, which, once processed by antigen-presenting cells (APCs), prime CD8^+^ and CD4^+^ T cells in the draining lymph nodes. These lymphocytes attack both primary tumor and metastatic sites, posing the biological basis of the *in situ* vaccination driving the so-called abscopal effect Ngwa et al. (10); Mole (11); Demaria et al. (12): RT induces a systemic behavior that can activate the immune response against metastasis, i.e., in locations that are far from the RT-treated primary tumor.

The involvement of the immune system has been demonstrated in different experimental models such as melanoma Twyman-Saint Victor et al. (13), colorectal Dovedi et al. (14) and breast cancers Demaria et al. (15), but the clinical presentation where considered anecdotal or at least rare.

Its rarity in clinical practice is likely due to the simultaneous engagement of immune escape mechanisms, such as the recruitment of regulatory CD4^+^ (Treg) and myeloid-derived suppressor cells counterbalancing the anti-tumor CD8^+^ T cell-mediated effects, and the tumor release of hypoxia-inducible factors with pro-survival activity Ji et al. (16).

More generally, susceptibility to the abscopal effect has been associated with several biological factors, such as tumor size or oxygen levels in tumor tissues. Indeed, the presence of hypoxic regions results in both increased resistance to the lethal effect of radiation mediated by reactive oxygen species (ROS) production and an immune suppressive tumor landscape ruled by Treg-recruiting chemokines and impaired APC function McNamee et al. (17); Castorina et al. (18)

Mathematical modeling approaches have become increasingly abundant in describing immunotherapy and its synergy with RT Dewan et al. (19) Agur and Vuk-PavlovićCheck that all equations and special characters are displayed correctly. (20) Walker and Enderling (21); Ng and Dai (22); Serre et al. (23) Gong et al. (24) Marconi et al. (25); Vanpouille-Box et al. (26); Chakwizira et al. (27); Kosinsky et al. (28); Liu et al. (29); Valentinuzzi et al. (30); Friedrich et al. (31); Malinzi et al. (32); Bekker et al. (33). Indeed, the complexity of cancer provides challenges and opportunities for new developments, and mathematical formulations contribute by helping to elucidate mechanisms and by quantitative predictions that can be validated experimentally Agur and Vuk-Pavlović (20); Altrock et al. (34); Brady and Enderling (35) .

A large part of mathematical models on tumor growth and therapies are based on sets of coupled differential equations. The number of equations increases together with the details of the biological description and this implies a large number of parameters and initial conditions to be specified. The detailed analyses are often so complex to require surrogate models for a reliable determination of the parameters Browning and Simpson (36).

On the other hand, more economical models introduce a macroscopic evolution of tumor growth and therapy with fewer parameters and a coarse-grain dynamical evolution Norton (2); Wheldon (37); Vaghi et al. (3); Guiot et al. (38); Castorina et al. (39); Castorina et al. (40).

This is the key point to obtain an effective quantitative control of the tumor progression. Indeed, microscopic models, which have the advantage of a deep understanding of the biological dynamics, of the physiologically-based pharmacokinetic [see for example Maaß et al. (41)] and of the possible translation to different populations/diseases, require many parameters and, although some of them can be determined by previous analyses, the parametric error propagation will produce a large band of fluctuation in the prediction of the quantitative evolution of the disease. Therefore we prefer to apply macroscopic growth laws of the sigmoid family with two parameters. Our choice of the GL is due to the result that untreated tumor growth has been better described by it (see Vaghi et al. (3) for a recent study). Moreover, in a transplantable rat tumor, it was shown that control and regrowth curves after radiotherapy could be fitted by the same Gompertzian law, provided adjustments for the initial lag and the estimated number of clonogens immediately after irradiation were performed [Jung et al. (42)]. Gompertzian growth has been assumed to describe human tumor repopulation during fractional radiotherapy also in Hansen et al. (43) and by O’Donoghue (44).

The main motivation of the proposed approach is, in our opinion, its complementary role in the clinical evaluation of disease progression, often based on macroscopic variables, and the better parameter identification, thus increasing model verifiability Braakman et al. (45).

Finally, this method offers a clear description of the complex interplay between radiotherapy, immunotherapy, and tumor progression, providing insights for advancing cancer treatment strategies that harness the abscopal effect.

The paper is structured as follows: the mathematical model, based on the GL and on the definition of the effective GL parameters, is recalled in the next section (Appendices A, B and C contain the corresponding calculations). Different macroscopic growth laws (as the LL) can be applied, without changing the underlying method. The emerging phenomenological approach, based on the suitable redefinition of the two GL parameters to describe the data, is reported in Section 3. The final sections are devoted to discussion and to the possible clinical use of the phenomenological model.

## 2 Methods

The proposed method is based on the mathematical model reported in detail in Appendices A, B and C in the **Supplementary Materials**. In what follows, the assumptions and some exact results are reported. Then, the phenomenological model is discussed.

### 2.1 Mathematical modeling

A general classification of macroscopic growth laws is reported in Castorina et al. (39) Castorina and Blanchard (46). For a population *N*(*t*) they are solutions of a general differential equation that can be written as


(1) $$\frac{1}{N(t)}\frac{dN(t)}{dt}=f[N(t)],$$

where *f*(*N*) is the specific growth rate and its *N* dependence describes the feedback effects during the time evolution. If in Equation 1 *f*(*N*) becomes constant, the growth follows an exponential pattern.

The untreated tumor progression is described by the GL Norton (2); Vaghi et al. (3), solution of the previous equation (see **Data Sheet 1**) with


(2) $$f\left[N(t)\right]=a-k\;\text{ln }\frac{N(t)}{N_{0}}=k\;\text{ln }\frac{N_{\infty }}{N(t)}$$

where *a,k,N*_0_ are constants that respectively indicate the exponential growth, the limiting factor, the initial cell number and *N*_∞_ is the carrying capacity (*N*_∞_ = *N*(0)*exp*(*a/k*)).

For untreated tumors, the GL emerges from microscopic, biological mechanisms where natural/adaptive immunity is taken into account Berendt and North (47); Gonzalez et al. (48); Castorina and Carco’ (49) The further effects of immune therapy, *I*(*t*), can be described by a modification of the previous Equation 2 as follows (see for example Wheldon (37))


(3) $$\frac{1}{N(t)}\frac{dN(t)}{dt}=k\text{ ln }\frac{N\infty }{N(t)}-\gamma \text{I}(t)$$

where *γ* is a constant.Notice that the sign of γ indicates the agonist or antagonist effect of the immune response: a negative *γ* increases the specific growth rate. The variable *I*(*t*) generically refers to the *passive* immunity resulting from the injection of anti-cancer-specific monoclonal antibodies i.e., the drug effects. However, it is, in general, unknown and requires a specific model. An example is the model of immunotherapeutic drug T11 target structure in the progression of malignant gliomas Khajanchi and Ghosh (50)Khajanchi and Banerjee (51).

The general solution of the previous equation and the IT effects on the tumor progression are discussed in **Mathematical Formalism**. For illustrative purposes, two specific cases are analyzed: *I*(*t*) = *I*(0) = *constant* and *I*(*t*) = *I*(0)*exp*(−*ρt*). The role of the therapy can be assimilated to a redefinition of an effective carrying capacity (in the first of the two cases) and, in general, in the introduction of effective, time and therapy dependent, parameters $a_{eff},k_{eff}\text{ or }N_{\infty }^{eff},k_{eff}$ , whose quantitative relation with *I*(*t*) is given in **Mathematical Formalism**. The introduction of an effective carrying capacity is well-known in population dynamics Royama (52). For example, the invention and diffusion of technologies lift the growth limit. Its possible time dependence is usually included by (at least) another differential equation, coupled with the growth equation. This will increase the number of parameters and initial conditions and, therefore, in our computational method the two effective parameters will be determined by data fits, giving a phenomenological indication about the disease progression.

There are different outcomes following an immune response to cancer Lin et al. (6): i) a surveillance role that inhibits the initiation and progression of the cancer; ii) the possibility that under certain conditions the immune response may nourish rather than curtail tumor growth. In other terms, monoclonal antibodies can exert antagonistic as well as sympathetic effects on tumor growth Lin et al. (6).

This possibility translates into a direct comparison among the parameters that describe the immunotherapy effects in Equation 3 and its solutions and the available data. For example, the determination of the crucial sign of the constant *γ*.

Let us now consider the combination of immune therapy (IT) and radiotherapy (RT). As a first step, we study the case of independent effects, i.e. no synergy between IT and RT.

The effect of radiotherapy is described by the linear quadratic model (LQM). Denoting by $N\left(t^{-}\right),N\left(t^{+}\right)$ respectively the cell number before and after the single dose *d* at time *t*, the RT effect is given by


(4) $$N\left(t^{+}\right)=N\left(t^{-}\right)e^{-D}=N\left(t^{-}\right)e^{-\alpha d-\beta d^{2}}$$

where *α* and *β* are constants (numerically *β* ≃ *α/*10) and *d* is the dose, Van Leeuwen et al. (53). The result in Equation 4 assumes an instantaneous effect of the RT, which could be, in general, not strictly applicable. Also, In this case, the therapy and immune response effects can be translated in the definition of effective parameters of the GL (see Mathematical Formulation).

The number of tumor cells after *n_f_* treatments at time *t_n_,t_n_*_+1_ *,t_n_*_+2_ *,…t_nf_* turns out to be (see **Mathematical Formalism**)


(5) $$N\left(t_{n_{f}}^{+}\right)=N(t_{0})e^{ln\frac{N_{\infty }}{N(t_{0})}[1-e^{-k(t_{n_{f}}-t_{0})}]-\bar{W}\left(t_{n_{f}},t_{0}\right)-D_{n_{f}}}$$

with the functions $D_{n_{f}}$ and $\bar{W}\left(t_{n_{f}},t_{0}\right)$ as described in **Mathematical Formalism**.

According to Equation 5, if


(6) $$ln\frac{N_{\infty }}{N(t_{0})}[1-e^{-k(t_{n_{f}}-t_{0})}]-\bar{W}\left(t_{n_{f}},t_{0}\right)-D_{n_{f}}<0$$

the tumor cell number decreases and the time evolution of the diseases moves toward complete recovery.

Therefore, although RT and IT are considered independent, if the effects of RT are such that


(7) $$ln\frac{N_{\infty }}{N(t_{0})}[1-e^{-k(t_{n_{f}}-t_{0})}]≃D_{n_{f}}$$

then, a small impact of the immunotherapy, $\bar{W}$ , can produce a tumor volume regression. Moreover, the critical conditions in Equations (6, 7) depend on the fractioning of the radiotherapy, since different schedules give different values of $D_{n_{f}}$ and $\bar{W}\left(t_{n_{f}},t_{0}\right)$ . Therefore, the previous conditions correspond to optimal control of the therapy effects, Khajanchi and Banerjee (54); Khajanchi (55); Khajanchi and Banerjee (56).

### 2.2 The synergy between immune and radio therapies

In the macroscopic framework, the description of the synergy between RT and IT requires a new term in the specific rate, which takes into account the immune response activated by RT, i.e.


(8) $$\frac{1}{N}\frac{dN}{dt}=kln(\frac{N_{\infty }}{N})-\gamma I(t)-\delta Y\left(t\right)F\left(d,t\right)$$

where *δ* is a constant, *F*(*d,t*) is a function of the dose *d* and of the time series of the treatments on the tumor, quantifying the cell-killing effect of the adaptive immune response *Y* (*t*), triggered by the RT. *Y* (*t*) is different from *I*(*t*) as it represents the outcome of the active immunization due to antigenic peptides coming from the disintegration of tissues hit by radiotherapy, and following the inflammation. To be more specific, *Y* (*t*) represents the immune response to tumor-associated antigens, promoted by the inflammation context due to the damage perpetrated by RT. Also, it must be specified that this immune response has a chance to exert an effect only before evasion mechanisms are established by the tumor (factors that are not counted in the present model).

If *d* = 0, *F*(*d,t*) = 0 there is no synergy. The specific form of the function *F*(*d,t*) requires a microscopic model, however one expects that the coupling *Y* (*t*)*F*(*d,t*), i.e. the immune activation due to RT, has a typical time decay, *τ*, after the single dose radiotherapy described by the LQM. For the primary tumor, the parameter *δ* is small, i.e., *δ <<* 1 and the synergy is small. The abscopal effect is described by considering a finite value of *δ* for metastases that are far away from the primary tumor location. The result for the time evolution of the abscopal effect is given in **Mathematical Formalism**.

## 3 Results

According to the mathematical approach in the previous section a phenomenological, simplified, method of analysis of the experimental data emerges. Indeed, a large part of the therapy and immune response effects can be assimilated to a redefinition of the GL parameters, with time and treatment dependence, which turns out to be detailed enough to compare with data. This phenomenological, simpler, procedure facilitates the validation of the model [Braakman et al. (45)] with respect to more complex analytical approaches, as discussed later.

Moreover, the function *I*(*t*)*,W*(*t*)*,Y* (*t*) in the previous differential equations are largely unknown, therefore the data fits of the effective GL parameters (see the general formulas in **Mathematical Formalism**) give model-independent information about the role of IT and RT.

### 3.1 Analysis of experimental data - immune therapy

**Figure 1:**
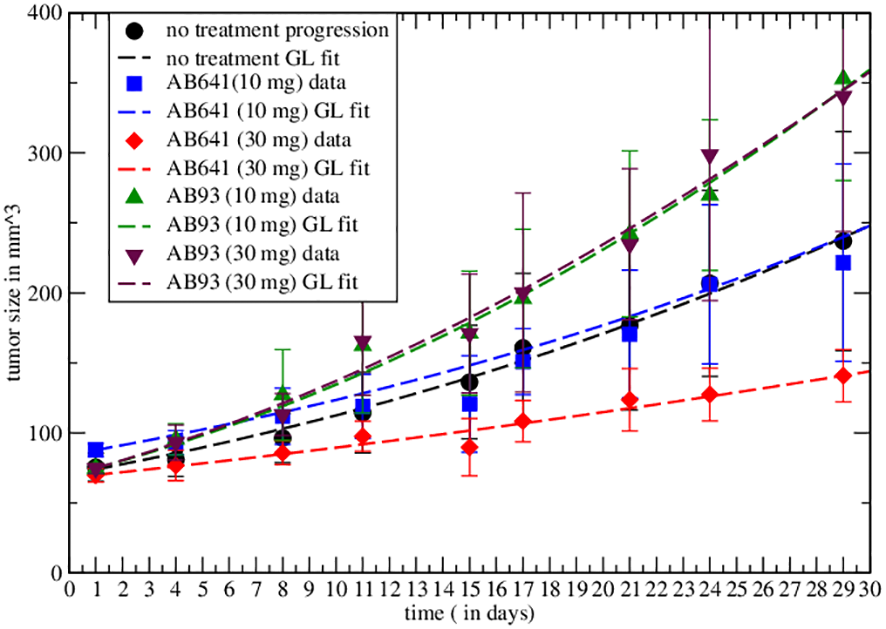
Comparison of the effective GL with data from the literature for untreated tumor and immunotherapy results. In the graph the symbols represent real data recorded and the dashed curves represent the Gompertzian fit, the colors are paired for both results. Fitted parameters are reported in **Table 1**.

In ref. Lin et al. (6) the different immune responses to cancer have been described. The authors highlight the agonist and antagonist effects of, respectively, AB93 and AB641 autoantibodies for the growth factor receptor TrkB in patients with breast cancer. After injection of MDA-MB-231 cells in immunodeficient mice they show the response to treatment, for different dosages of autoantibodies, measuring tumor growth. In particular, the data on the effects of AB641 and AB93 on tumor progression can be analyzed in the proposed macroscopic approach by Equation 3, by redefining GL parameters. Initially, one has to fit the untreated tumor progression data by GL to determine the corresponding parameters. Then, one repeats the analysis with immunotherapy. The results are shown in **Figure 1**, which clearly reveal the agonist or antagonist role of the different antibodies.

The experimental result in the case of therapy can be fitted by redefining the GL parameters with respect to the untreated ones (see **Equation S2**, **Equation S23** and **Mathematical Formalism** in **Supplementary Material**). The values are reported in **Table 1**. The agonist effects increase both parameters, producing faster growth, corresponding to a negative *γ * in Equation 3, whereas the antagonistic effect induces tumor depletion. Notice that the change in the GL parameters, i.e. of the therapy, implies a modification of the exponential rate and the carrying capacity with respect to the untreated tumor.

**Table 1:** GL effective parameters of the comparison in **Figure 1** (see **Equation S23** in **Supplementary Material**.

Monoclonal antibody	*a_eff_*	*k_eff_*
AB641 (10 mg)	0.0285 ± 0.0037	0.0096 ± 0.0012
AB641 (30 mg)	0.034 ± 0.0054	4.7 ∗ 10^−5^ ± 1.1 ∗ 10^−6^
Untreated	0.049 ± 0.0008	0.011 ± 0.00016
AB93 (10 mg)	0.0714 ± 0.0069	0.02 ± 0.0016
AB93 (30 mg)	0.075 ± 0.0085	0.024 ± 0.002

Statistical error based on χ^2^ per degree of freedom. Parameters in day^−1^.

Non-linear curve fitting was made using Grace (version 5.1.25) Dataset Grace (57), data fitted by GL effective parameters are promising. The correlation coefficients and the root mean squared relative errors are respectively given by (0.982,0.062),(0.977,0.052),(0.995,0.051),(0.992,0.058),(0.996,0.04) for AB641/30 (red curve in **Figure 1**), AB641/10 (blue), AB93/10 (green), AB93/30 (black triangle) and untreated case.

### 3.2 Analysis of experimental data - RT and abscopal effect

The abscopal effect has been experimentally studied in Nesseler et al. (58) by inoculation of undifferentiated fibrosarcoma cells (FSA1) into immunocompetent mice to simulate primary and metastatic conditions, successively divided in four treatment groups: no treatment, anti-PD-1 monoclonal antibody alone, RT alone, and combination of anti-PD-1 with RT.

Initially, only the effect of RT on the primary has been detected, showing a critical dose administration for tumor regression [**Figure 2A** of ref. Nesseler et al. (58)]. The limited role of anti-PD-1 on the untreated primary tumor has been observed [**Figure 2B** of ref. Nesseler et al. (58)] and it has been checked that the RT on the primary has no direct effect on the implanted secondary. Finally, the synergy between IT and RT has been verified by the regression of the metastasis.

**Figure 2:**
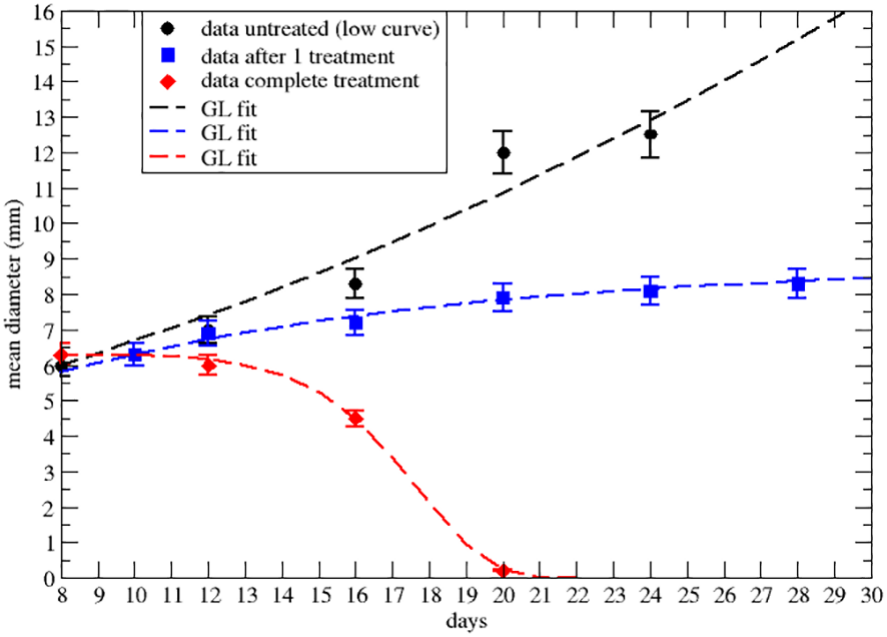
Effective GL fit of literature data with effective parameters of the lower data set of **Figure 2A** of ref.Nesseler et al. (58). Black point and the dashed line represent respectively real data and Gompertzian fitting. Blue squares and curve are data and GL fit after the first 8Gy RT treatment. The red rhombus and curve are data and GL after three treatments of 8Gy each. Parameters reported in **Table 2**.

**Figure 3:**
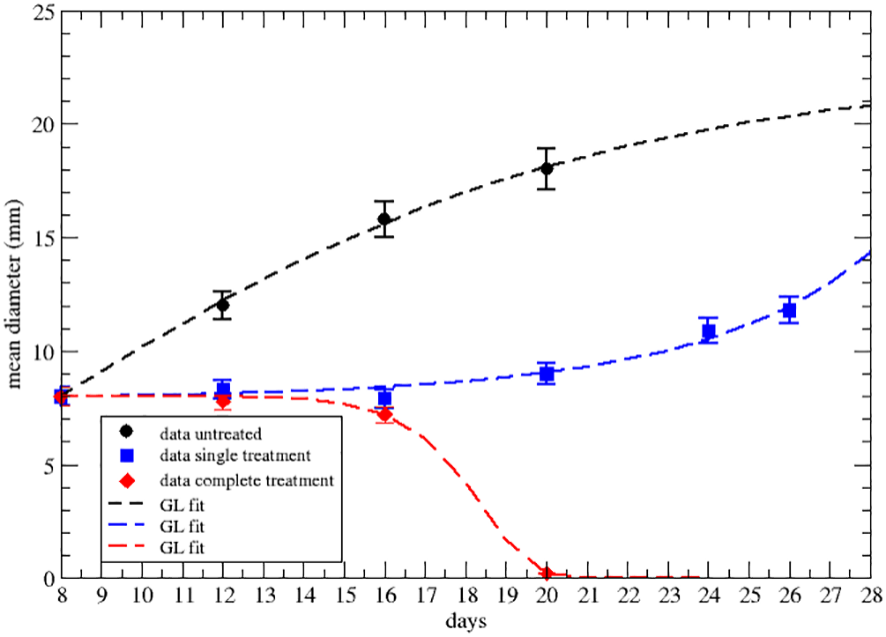
GL fit of literature data with effective parameters of the upper data set of **Figure 2A** of ref.Nesseler et al. (58), where the untreated data are in black, blue for one shot of 8Gy of irradiation and red for three 8Gy shots. Parameter reported in **Table 3**.

Let us first consider the data on the primary tumor, treated by RT only, with three treatments of 8 Gy on days 9,10 and 11 after the injection of the cancer cells. The qualitative analysis [**Figure 2A** of ref. Nesseler et al. (58)] clearly indicates that the LQM with instantaneous cell killing effect is not able to reproduce the observed effect, due to a delay between the treatments (on days 9,10 and 11) and the regression behavior (i.e., a negative specific rate), starting on day 15, Lim et al. (59); McMahon (60). Therefore, more complex dynamics are in place, which, however, can still be described by a GL pattern. As discussed, the effective parameters can be time-dependent (see **Equation S23**-**S30** in **Mathematical Formalism**) due to the therapy and the results are reported in **Figures 2**, **3** respectively for the lower and upper data sets of **Figure 2A** of ref. Nesseler et al. (58). The corresponding fitted effective parameter values are given in **Tables 2**, **3**. The (∗) indicates that the fitted parameter *a_eff_* has a linear time dependence *a_eff_* → *a_eff_* (*t* − *t*_0_). This time dependence and the sign change of *k_eff_*, compared to the untreated case, signal that the therapy produces a complete depletion of the tumor size, analogously to the extinction in population dynamics.

**Table 2:** GL effective parameters of the comparison in **Figure 2** (see **Equation S23** in **Supplementary Material**).

Tumor size	*a_eff_*	*k_eff_*
untreated	0.054 ± 0.0031	0.0164 ± 0.0015
after first dose	0.036 ± 0.0058	0.11 ± 0.017
end of therapy (∗)	−5.06 ∗ 10^−4^ ± 2 ∗ 10^−6^	−0.464 ± 0.003

Parameters in day^−1^. Statistical error based on χ^2^ per degree of freedom.

**Table 3:** GL effective parameters of the comparison in **Figure 3** (see **Equation S23** in **Supplementary Material**.

Tumor diameter (mm)	*a_eff_*	*k_eff_*
untreated	0.135 ± 0.011	0.131 ± 0.11
after first dose	2.82 ∗ 10^−3^ ± 2 ∗ 10^−4^	−0.184 ± 0.015
end of therapy (∗)	−1.99 ∗ 10^−5^ ± 6 ∗ 10^−7^	−0.783 ± 0.002

Parameters in day^−1^. Statistical error based on χ^2^ per degree of freedom.

**Figure 4:**
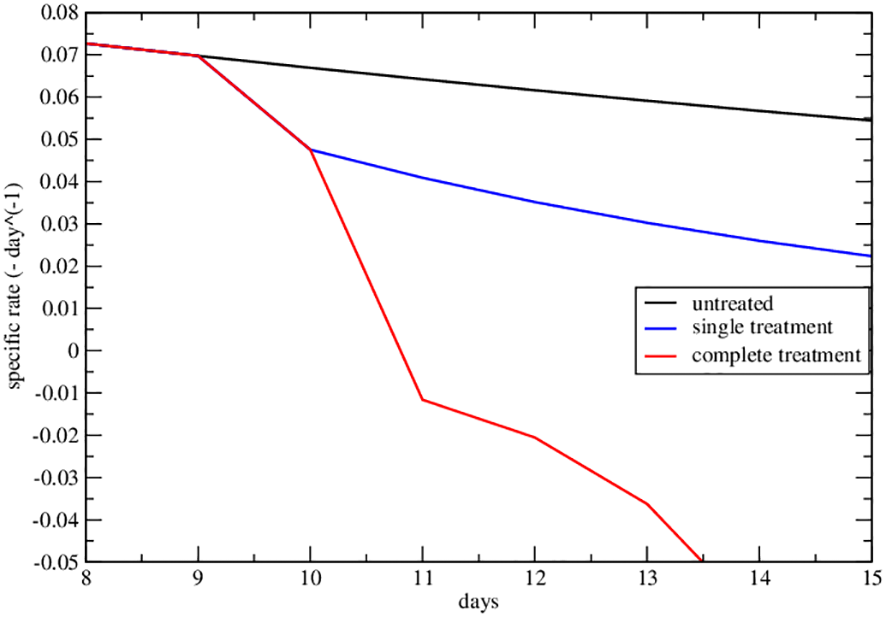
Specific rate of the lower data set of **Figure 2A** of ref. Nesseler et al. (58). The curves represent the specific growth rate day by day, the response to therapy is highlighted by a negative trend.

**Figure 5:**
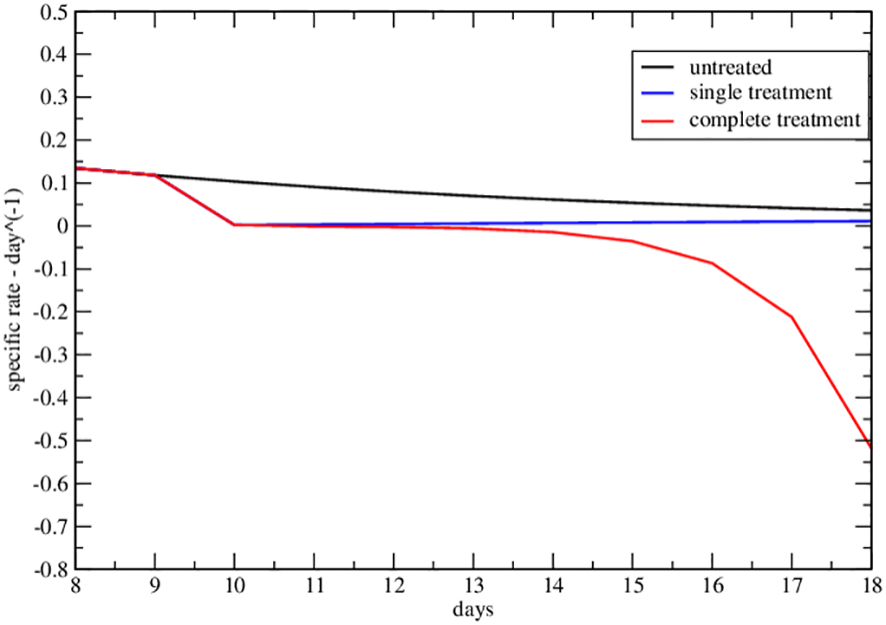
Specific rate of the upper data set of **Figure 2A** of ref. Nesseler et al. (58). The curves represent the specific growth rate day by day, the response to therapy is highlighted by a negative trend.

The strong signal of the change of sign in the effective parameters can also be summarized by plotting the specific rate (1*/NdN/dt*), in **Figures 4**, **5**, where its negative value indicates the complete tumor regression trend.

It should be stressed that the approach with GL effective parameters, suggested by the more rigorous previous mathematical model (see **Mathematical Formalism**), is a phenomenological one with the aim of a simplified clinical, but quantitative, understanding of the tumor progression at a more personalized level (see next discussion section).

The final analysis concerns the abscopal effect, according to Equation 8 with *γ* = 0. Let us assume: a) an exponential decay of the immune response with a time delay *τ* between the RT on the primary and the immunological effect on the secondary; b) the activated immune system continues its effect on the secondary with an exponential rate corresponding to the specific rate obtained at the end of the RT.

In other words, if *t_in_* is the starting day of RT on the primary, for *t < t_in_* the metastatic site evolves according to the GL progression with the untreated parameters. At *t_in_* the RT starts and the immune system targets the secondary (see Equation 8 and its solution reported in **Mathematical Formalism**). At the end of the RT, the immunity response continues to reduce the metastasis with an exponential behavior if the specific rate turns out to be negative.

**Figure 6:**
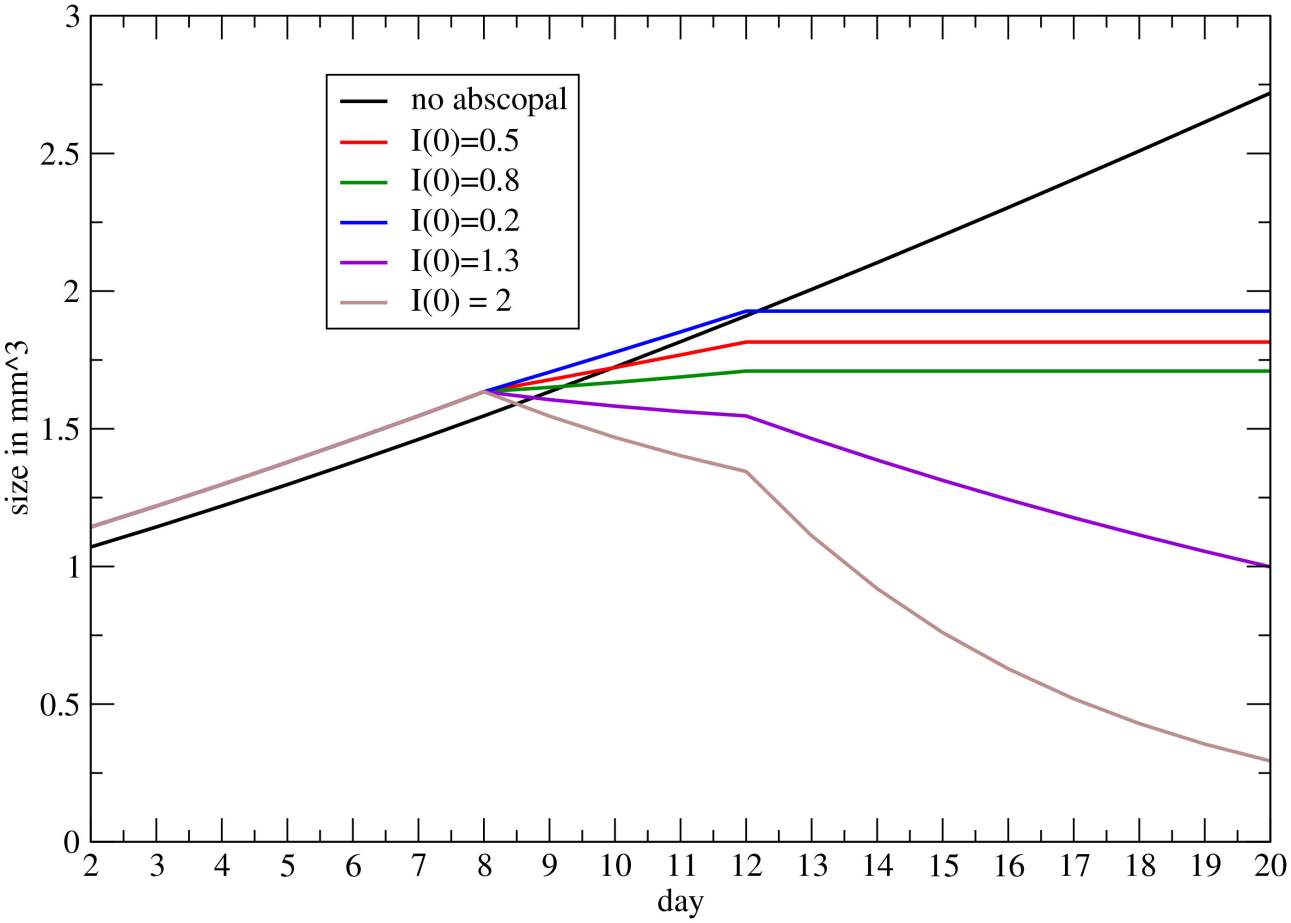
Progression on the metastatic volume, triggered by RT on the primary, for different values of the coupling between the immune system and RT (see **Mathematical Formalism**).

In **Figure 6** the result is depicted, i.e., the abscopal effect, according to the previous approach, for different values of the parameter *I*(0), determining the response of the immune system on the secondary induced by RT (see **Mathematical Formalism**).

## 4 Discussion

The phenomenological approach, based on the previous mathematical formulation, consists in fitting the specific rate data by GL with effective parameters. Let us recall that the specific rate is much more reliable than the volume tumor variation, in determining its progression and the phase of growth or decrease.

We are aware that the coarse-grain proposed approach misses the detailed dynamics and can be considered an oversimplified description since the underlying pathways, Ng and Dai (22); Serre et al. (23); Marconi et al. (25); Liu et al. (29); Valentinuzzi et al. (30); Friedrich et al. (31); Bekker et al. (33) are summarized by the macroscopic Equation 8. However, one has to recall that, independently of the microscopic conditions, a large part of untreated tumors follow the GL (see Vaghi et al. (3) for a recent review) and that with a small number of parameters, one gets quantitative clinical indications for personalized treatments. If, for example, a patient gets a small specific rate by RT on the primary tumor, then a small contribution of IT might be able to result in a complete recovery.

The abscopal effect has been described by a macroscopic coupling between RT and immune system response, where the initial progression of the metastasis follows the GL with untreated primary tumor parameters. This is a reasonable assumption although the in-situ conditions can produce different results.

According to the specific conditions recalled in the introduction, choosing the best dose fractionation and timing with respect to immunotherapy is difficult. Notoriously, the use of protracted RT schedules (standard fractionation or slight hypofractionation) is discouraged since radiosensitive lymphocytes are cleared out from tumor tissues at each fraction delivery, thus preventing their anti-tumor function, Filatenkov et al. (61). Conversely, large doses per fraction are effectively immunogenic, Muraro et al. (62). In particular, doses below 12 Gy are the most suitable for enhancing the anti-tumor immune response as over such a threshold there is the degradation of immunogenic cytosolic DNA by an exonuclease, Trex, whose expression, as evaluated in preclinical experiments, is cell line-dependent and increases with increasing radiation dose Dewan et al. (19); Vanpouille-Box et al. (26). On the other side, even very low doses per fraction (*<* 1 Gy) seem to activate macrophages against cancer cells and stimulate T-cell immunity Klug et al. (63). Doses over 12 Gy are involved in the damage of tumor vasculature by activation of acid sphingomyelinase and production of ceramides, which culminate in vessel obliteration with subsequent tumor regression for insufficient nutrient and oxygen supply Song et al. (64). Therefore, the entire dose range used in clinical practice may be useful to control tumors and all RT fractionations combined with immunotherapy deserve clinical investigations.

Recently, old RT techniques simultaneously combining very different doses within the tumor, namely spatially fractionated radiation therapy (SFRT), are gaining new interest because of the assumption that tumor tissues spanning a wide dose range may benefit from multiple immune activation mechanisms, which eventually could be further boosted by ICI administration Ferini et al. (65); Tubin et al. (66). Given the ability of new instrumental exams to “map” tumor areas with different metabolisms, there is the possibility of modulating the dose distribution according to the oxygenation patterns inside the tumor to maximize both direct and indirect (immune-mediated) lethal effects of radiation Ferini et al. (67); Ferini et al. (68). With the latter approach, complete responses have been documented earlier than with classic homogeneously-delivered stereotactic RT doses and before ICI administration, likely implying rapid immune intervention enhanced and maintained by the addition of IT Ferini et al. (69).

All of the above considerations require the modeling of tumor response to RT and IT to help predict the best combination strategy, also given the inadequacy of current radiobiological mathematical models to comprehensively explain the results deriving from this association Ferini et al. (69).

In the proposed mathematical and computational approach, the fractionization effects are taken into account by the functions $D_{n_{f}}$ and $\bar{W}\left(t_{n_{f}},t_{0}\right)$ and a spatially non-homogeneous behavior can be easily implemented. However, a complete discussion requires a forthcoming devoted analysis.

## 5 Conclusions

A comprehensive scope of the combined impact of radiotherapy and immunotherapy is vital for clinical decision-making. Yet, numerous mathematical models in existing literature prove overly complex, characterized by an abundance of differential equations, parameters, and initial conditions, rendering their practical implementation quite challenging.

In this study, we use a macroscopic mathematical approach that does not rely on the underlying microscopic dynamics. Instead, we propose a simplified model of the tumor progression using a Gompertz law, which involves just two parameters. Moreover, utilizing numerical solvers of the equations provided in the appendices is a straightforward process.

Examining how control influences system dynamics under radiation and/or drug therapy sheds light on the disease’s temporal progression. This approach holds promise in assisting clinicians to make informed decisions by providing a clearer understanding of treatment outcomes, namely, assessing whether the therapy administered results in full recovery.

Besides its clinical relevance, our approach shows potential for additional experimental validation of the synergistic impact of the abscopal phenomenon on treatment outcomes.

We recognize the importance of conducting rigorous experimental investigations to solidify the theoretical basis of our approach. By performing targeted studies and gathering more empirical data, we aim to validate the approach in different conditions and the significance of the abscopal effect in influencing therapy outcomes. This experimental validation will provide a deeper understanding of the interplay between the administered treatment, tumor response, and the abscopal effect.

Moreover, our future research endeavors will focus on elucidating the mechanisms underlying the abscopal effect and quantifying its impact on treatment efficacy. By combining computational modeling with comprehensive experimental studies, we strive to enhance our understanding of this phenomenon and optimize therapeutic strategies accordingly. Ultimately, we aim to improve patient-oriented outcomes by harnessing the full potential of the abscopal effect in cancer therapy.

## Data availability statement

Publicly available datasets were analyzed in this study. This data can be found here: https://figshare.com/articles/dataset/autoantibody_discovery/10302227; data on AB93 and AB641, platform figshare.

## Author contributions

PC: Conceptualization, Formal analysis, Methodology, Writing – original draft. FC: Conceptualization, Data curation, Investigation, Validation, Writing – review & editing. GF: Methodology, Validation, Writing – original draft. SF: Funding acquisition, Methodology, Resources, Validation, Writing – review & editing. EM: Data curation, Investigation, Software, Validation, Writing – review & editing. DG: Funding acquisition, Resources, Supervision, Writing – review & editing.

## Supplementary material

Mathematical Formalism Mathematical model for untreated tumor growth with GL, immune therapy, immune and radiotherapies, critical behavior, and abscopal effect.
