# Supplementary material for: Mathematical modeling of the synergistic interplay of radiotherapy and immunotherapy in anti-cancer treatments
Source: Front Immunol. 2024 May 8;15:1373738. doi: 10.3389/fimmu.2024.1373738 (PMC11109403; doi:10.3389/fimmu.2024.1373738)
Supplement: Mathematical Formalism — Mathematical model for untreated tumor growth with GL, immune therapy, immune and radiotherapies, critical behavior, and abscopal effect. [file DataSheet_1.pdf]

Supplementary Material

1 APPENDIX A

An untreated tumor grows according to the Gompertz law (GL), solution of the equation

$$\frac{1}{N}\frac{dN}{dt} = kln(\frac{N_\infty}{N}) \tag{S1}$$

where N is the number of cells, k is a constant with dimension $(\text{time})^{-1}$ and N_∞ is the carryimg capacity, i.e. the maximum number of cell that the boundary condition of the growth can support. The GL, solution of eq.(S1), is given by

$$N(t) = N(t_0)e^{ln\frac{N_\infty}{N(t_0)}[1-e^{-k(t-t_0)}]}. \tag{S2}$$

The equation is then modified to take into account the effects of the therapy (radio, immune, chemo - therapy).

1.1 Immune therapy

Let us first consider the role of the immune therapy $I(t)$, i.e. let us modify the specific growth rate (the second term in eq.S1) according to

$$\frac{1}{N}\frac{dN}{dt} = kln(\frac{N_\infty}{N}) - \gamma I(t) \tag{S3}$$

where γ is a constant and the variable $I(t)$ generically refers to the *passive* immunity resulting from the injection of anti-cancer specific monoclonal antibodies.

The solution of eq.S3 is

$$N(t) = N(t_0)e^{ln\frac{N_\infty}{N(t_0)}[1-e^{-k(t-t_0)}]-\gamma\int_{t_0}^{t}dt^{'}I(t^{'})e^{-k(t-t^{'})}} \tag{S4}$$

The time dependence of $I(t)$ is unknown and requires a microbioloical model. Therefore, let us discuss two useful examples. First let us assume $I(t) = I(t_0) = constant$, therefore one gets

$$\gamma\int_{t_0}^{t}dt^{'}I(t^{'})e^{-k(t-t^{'})} = \frac{\gamma I(t_0)}{k}[1-e^{-k(t-t_0)}] \tag{S5}$$

which implies that the effect of a constant immune activity can be reabsorbed by a redefinition of the term $ln(N_\infty)/N(t_0)$, i.e. of the parameters a, k, as shown in appendix B. Let us consider a exponential behavior, i.e. $I(t) = I_(0)exp(-\rho t)$. In this case, the correction to the untreated growth turns out to be

$$\gamma\int_{t_0}^{t}dt^{'}I(t^{'})e^{-k(t-t^{'})} = \frac{\gamma I(t_0)}{k-\rho}e^{-\rho t}[1-e^{-(k-\rho)(t-t_0)}] \tag{S6}$$

which is not a simple redefinition of the carrying capacity, but rather suggests an effective, time-dependent, redefinition of the two constants k and N_∞ (see appendix B)

Notice that if the immune therapy is activated after some time t_a, i.e. the function $I(t)$ contains a Θ-function, $\Theta(t - t_a)$, there is no effect for $t < t_a$.

1.2 Immune and Radio therapies - independent effects

The effect of radiotherapy is described by the linear quadratic model (LQM). Denotin by $N(t^-)$ the cell number before the single dose d at time t, the number of cells after the treatment is given by

$$N(t^+) = N(t^-)exp(-D) = N(t^-)exp(-\alpha d - \beta d^2) \tag{S7}$$

where α and β are constants (usually $\beta = \alpha/10$).

Let us consider that, starting at time t_n, one has a series of n single treatments at time $t_n < t_{n+1} < t_{n+2}..... < t_{n_f}$. Before the first dose, including the immune response ($I(t)$), one has

$$N(t_n^-) = N(t_0)e^{ln\frac{N_\infty}{N(t_0)}[1-e^{-k(t_n-t_0)}]-W(t_n,t_0)} \tag{S8}$$

where

$$W(t_n, t_0) = \gamma \int_{t_0}^{t_n} dt^{'} I(t^{'})e^{-k(t_n-t^{'})}. \tag{S9}$$

Let us recall that if the immune response due to the therapy has not been activated during the considered time interval, then$I(t) = 0$.

After the single dose one has $N(t_1^+) = N(t_1^-)exp(-D)$.

By iteraction, one can show that after n_f dose one gets

$$N(t_{n_f}^+) = N(t_0)e^{ln\frac{N_\infty}{N(t_0)}[1-e^{-k(t_{n-f}-t_0)}]-\bar{W}(t_{n-f},t_0)-D_{n-f}} \tag{S10}$$

where

$$\bar{W}(t_{n_f}, t_0) = W(t_{n_f}, t_0) - \Sigma_{i=1}^{n_f} W(t_{i-1}, t_0)[1 - e^{-k(t_i - t_{i-1})}]e^{-k(t_{n_f}-t_i)} \tag{S11}$$

and

$$D_{n_f} = (\alpha d + \beta d^2)\Sigma_{i=0}^{n_f} e^{-k(t_{n_f}-t_i)} \tag{S12}$$

For regular time interval of dose administration, Δt, then $t_n = n\Delta t$ and $t_i = i\Delta t$ and, by resummation, one gets

$$D_{n_f} = (\alpha d + \beta d^2)\frac{1 - e^{-(t_{n_f}/\Delta t)}}{1 - e^{-k\Delta t}}. \tag{S13}$$

1.3 Critical behavior

The previous equations apply to the primary tumor and the abscopal effect will be discussed in the next section. According to clinical results, the immunotherapy effect on the primary tumor is small. However, it can produce important changes in the progression if, for example, radiotherapy is able to compensate for the growth rate. More precisely, eq.(S10) shows that $N(t_{n_f}^+) < N(t_0)$ if

$$ln\frac{N_\infty}{N(t_0)}[1 - e^{-k(t_{n_f}-t_0)}] - \bar{W}(t_{n-f}, t_0) - D_{n_f} < 0. \tag{S14}$$

Therefore, if, for example, the radiotherapy effects are such that

$$ln\frac{N_\infty}{N(t_0)}[1-e^{-k(t_{n_f}-t_0)}] \simeq D_{n_f}, \tag{S15}$$

also a small effect due to the immunotherapy, $\bar{W}(t_{n-f}, t_0)$, can produce a tumor volume regression. Moreover, the critical condition depends on the fractionization, since different radiotherapy schedules give different values of D_{n_f} and $\bar{W}(t_{n-f}, t_0)$.

1.4 Abscopal effect

The abscopal effect, i.e. the immune response, triggered by RT on the primary, on a distant secondary progression, requires an explicit, non-linear, term in the previous equations. In particular, for the cell population in the secondary, one can write

$$\frac{1}{N_s}\frac{dN_s}{dt} = k_s ln(\frac{N^s_\infty}{N_s}) - \delta I_s(t)F(d,t) \tag{S16}$$

where N_s is the cell number, k_s, N^s_∞ are the corresponding GL parameters, δ is a constant, I_s is the immune response on the metastasis and $F(d,t)$ is, in general, an unknown function of the dose d and and on the time series of the treatments on the primary tumor. The determination of $F(d,t)$ requires a microbiological model, but if $d = 0$, $F(d,t) = 0$ and the factor $I_s(t)F(d,t)$ has to take into account that the immune activation as a typical time decay, τ after the administration of a single dose radiotherapy, described by the LQM. A possible choice is

$$I_s(t) = I_s(0)e^{-(t_i/\tau)}. \tag{S17}$$

Then according to the scheduled treatments at time $t_n < t_{n+1} < t_{n+2}..... < t_{n_f}$, before the first dose (i.e. at time t_n^-) the cell number is given by

$$N_s(t_n^-) = N_s(t_0)e^{ln\frac{N_\infty}{N_s(t_0)}[1-e^{-k(t_n-t_0)}]} \tag{S18}$$

and then at the end of the time evolution $t_n \to t_{n+1}^-$ one gets ($\lambda = 1/\tau$)

$$N_s(t_{n+1}^-) = N_s(t_0)e^{ln\frac{N_\infty}{N_s(t_0)}[1-e^{-k(t_{n+1}-t_0)}]-\delta(\alpha d+\beta d^2)I_s(t_n)e^{-kt_{n+1}}\int_{t_n}^{t_{n+1}}dt' e^{(k-\lambda)t'}}, \tag{S19}$$

that is

$$N_s(t_{n+1}^-) = N_s(t_0)e^{ln\frac{N_\infty}{N_s(t_0)}[1-e^{-k(t_{n+1}-t_0)}]-\delta I_s(t_n)\frac{\alpha d+\beta d^2}{k-\lambda}e^{-\lambda t_{n+1}}[1-e^{-(k-\lambda)(t_{n+1}-t_n)}]}. \tag{S20}$$

where $I_s(t_n)$ is the initial activation of the immune system due to RT. The time evolution can iteratively be evaluated by the general formula

$$N_s(t_i) = N_s(t_{i-1})e^{ln\frac{N_\infty}{N(t_{i-1})}[1-e^{-k(t_i-t_{i-1})}]-\delta(\alpha d+\beta d^2)I_s(t_{i-1})e^{-kt_i}\int_{t_{i-1}}^{t_i}dt' e^{(k-\lambda)t'}}, \tag{S21}$$

for $i > 2$.

2 APPENDIX B. EFFECTIVE PARAMETERS

The definition of the effective parameters is given in this appendix.

2.1 Immune Therapy

Let us start with the case $I(t) =$ constant, i.e. from eqs.(S4,S5) of Appendix A. The solution is given by

$$N(t) = N(t_0) e^{ln\frac{N_\infty}{N(t_0)}[1-e^{-k(t-t_0)}] - \frac{\gamma I(t_0)}{k}[1-e^{-k(t-t_0)}]} \tag{S22}$$

By defining

$$N(t) = N(t_0) e^{ln\frac{N_\infty^{eff}}{N(t_0)}[1-e^{-k_{eff}(t-t_0)}]}, \tag{S23}$$

in this specific case one gets

$$N_\infty^{eff} = N_\infty e^{-\frac{\gamma I(t_0)}{k}} \tag{S24}$$

and

$$k_{eff} = k \tag{S25}$$

For the exponential behavior, eqs.(S4,S6) of Appendix A, the solution is

$$N(t) = N(t_0) e^{ln\frac{N_\infty}{N(t_0)}[1-e^{-k(t-t_0)}] - \frac{\gamma I(t_0)}{k-\rho} e^{-\rho t}[1-e^{-(k-\rho)(t-t_0)}]}. \tag{S26}$$

By the same definitions in eq.(23), after some algebra, one gets effective time dependent parameters given by:

$$N_\infty^{eff} = N_\infty e^{-\frac{\gamma I(t_0)}{k-\rho} e^{-\rho t}} \tag{S27}$$

and k_{eff} defined by the following equation

$$e^{-k_{eff}(t-t_0)} = \frac{\gamma I(t_0)}{k-\rho} \frac{1}{ln[N_\infty^{eff}/N(t_0)]} e^{-k(t-t_0)} \tag{S28}$$

2.2 General Case

In general, if the solution is given by

$$N(t) = N(t_0) e^{ln\frac{N_\infty}{N(t_0)}[1-e^{-k(t-t_0)}] - \gamma \int_{t_0}^{t} dt' F(t') e^{-k(t-t')}} \tag{S29}$$

with $\gamma =$ constant and $F(t)$ function of time, the definition of the effective parameters by eq.(23) turns out to be

$$N_\infty^{eff} = N_\infty e^{-\gamma \int_{t_0}^{t} dt' F(t') e^{-k(t-t')}} \tag{S30}$$

and

$$k_{eff} = k + \frac{1}{t-t_0} ln[1 - \frac{\gamma}{lnN_\infty/N(t_0)} \int_{t_0}^{t} dt' F(t') e^{-k(t-t')}] \tag{S31}$$

3 APPENDIX C

This appendix contains the proof of formulas S10-S12. The solution of eq.(S3) is

$$ln\frac{N(t)}{N(t_0)} = ln\frac{N_\infty}{N(t_0)}[1-e^{-k(t-t_0)}] - \gamma\int_{t_0}^{t} dt^{'} I(t^{'})e^{-k(t-t^{'})} \text{(S32)}$$

By considering n single treatments at time $t_n < t_{n+1} < t_{n+2}..... < t_{n_f}$, after the first radiotherapy dose at time t_n (time in unit of the treatment interval Δt; the time will be indicated by $\pm$, referering to before , $-$, or after , $+$ the single treatment). Therefore one gets

$$N(t_n^+) = N(t_n^-)exp(-\alpha d - \beta d^2) \quad \text{(S33)}$$

By previous eqs.(32,33), the number of cells before the second treatment (i.e. at t_{n+1}^-) is given by

$$ln\frac{N(t_{n+1}^-)}{N(t_n^+)} = ln\frac{N_\infty}{N(t_n^+)}[1-e^{-k\Delta t}] - \gamma\int_{t_n}^{t_{n+1}} dt^{'} I_{n+1,n}(t^{'})e^{-k(t_{n+1}-t^{'})}.\text{(S34)}$$

where $I_{n+1,n}$ indicates the function $I(t)$ in the time interval (t_n, t_{n+1}). Since

$$N(t_{n+1}^+) = N(t_{n+1}^-)exp(-\alpha d - \beta d^2), \quad \text{(S35)}$$

by eq.(S34) one has

$$N(t_{n+1}^+)/N(t_n^-) = exp(T_{n+1}^+) \quad \text{(S36)}$$

where

$$T_{n+1}^+ = ln\frac{N_\infty}{N(t_n^-)}[1-e^{-k\Delta t}] - \gamma\int_{n}^{n+1} dt^{'} I_{n+1,n}(t^{'})e^{-k(t_{n+1}-t^{'})} - (\alpha d + \beta d^2)[1+e^{-k\Delta t}] \quad \text{(S37)}$$

The evolution from t_{n+1}^+ to t_{n+2}^- is given by

$$ln\frac{N(t_{n+2}^-)}{N(t_{n+1}^+)} = ln\frac{N_\infty}{N(t_{n+1}^+)}[1-e^{-k\Delta t}] - \gamma\int_{n+1}^{n+2} dt^{'} I_{n+2,n+1}(t^{'})e^{-k((n+2)-t^{'})}.\text{(S38)}$$

By substitution of $N(t_{n+1}^+)$, one obtains $N(t_{n+2}^+)/N(t_n^-) = exp(T_{n+2}^+)$ with

$$T_{n+2}^+ = ln\frac{N_\infty}{N(t_n^-)}[1-e^{-2k\Delta t}] - \gamma e^{-k\Delta t}\int_{n}^{n+1} dt^{'} I_{n+1,n}(t^{'})e^{-k((n+1)-t^{'})} \quad \text{(S39)}$$

$$-\gamma\int_{n+1}^{n+2} dt^{'} I_{n+2,n+1}(t^{'})e^{-k((n+2)-t^{'})} - (\alpha d + \beta d^2)[1+e^{-k\Delta t}+e^{-(n+1)k\Delta t}]$$

By iteraction, the final result is $N(t_{n_f}^+)/N(n^-) = exp(T_{n_f^+})$ with

$$T^{+}_{n_f} = ln\frac{N_\infty}{N(t_n^-)}[1 - e^{-(n_f-n)k\Delta t}] - (\alpha d + \beta d^2)\Sigma_{i=n}^{n_f} e^{(i-n)k\Delta t} \tag{S40}$$

$$-\gamma\Sigma_{i=n}^{n_f-1} e^{-(n_f-i)k\Delta t}\int_i^{i+1} dt^{'} I_{i+1,i}e^{-((i+1)-t')} \tag{S41}$$

$$-\gamma(1 - e^{-k\Delta t})\int_{n_f-1}^{n_f} dt^{'} I_{n_f,n_f-1}e^{-(n_f-t')}$$

By definition of $W(i+1,i)$ (see eq.9) one gets

$$T^{+}_{n_f} = ln\frac{N_\infty}{N(t_n^-)}[1 - e^{-(n_f-n)k\Delta t}] - (\alpha d + \beta d^2)\Sigma_{i=n}^{n_f} e^{(i-n)k\Delta t} \tag{S42}$$

$$-\gamma\Sigma_{i=n}^{n_f-1} e^{-(n_f-i)k\Delta t} W(i+1,i) - \gamma(1 - e^{-k\Delta t})W(n_f, n_f - 1)$$
